# Supplementary material for: Circuits that encode and guide alcohol-associated preference
Source: eLife. 2020 Jun 4;9:e48730. doi: 10.7554/eLife.48730 (PMC7272191; doi:10.7554/eLife.48730)
Supplement: Supplementary file 1. — Table 1 includes a cell count of PAM neuron driver lines. Table 2 includes odor sensitivity controls for all memory experiments performed. Table 3 includes a description of target and off-target expression of split-Gal4 lines used. Table 4 includes a comprehensive table of detailed statistics that describe all data. Table 5 includes a review of papers published that include use of the RNAi lines used here. [file elife-48730-supp1.docx]

**Circuits that encode and guide alcohol associated preference**

Kristin M. Scaplen^1^, Mustafa Talay^1,5^, Kavin Nunez^3,^ Sarah Salamon^2^, Amanda Waterman^1^, Sydney Gang^4^, Sophia Song^1^, Gilad Barnea^1^, Karla R. Kaun^1^*

^1^ Department of Neuroscience, Brown University, Providence RI 02912

^2^ Department of Pharmacology, University of Cologne, Cologne, Germany

^3^ Department of Molecular Pharmacology and Physiology, Brown University, Providence, RI, USA

^4^ Department of Biochemistry, Brown University, Providence, RI, USA

^5^ Current address: Howard Hughes Medical Institute, Department of Molecular and Cellular Biology, Harvard University, Cambridge, USA

* Corresponding author: [karla_kaun@brown.edu](mailto:karla_kaun@brown.edu)

Contents:

Supplemental Table 1………....…… ……………………………………….…………………..2

Supplemental Table 2 .………………… …………………………..…………………………...3

Supplemental Table 3 …………………………………………………………………………....4

Supplemental Table 4 …………………… ……………………..…………………………...5-12

Supplemental Table 5………………………………………………………………………...…13

Supplemental References .………………………………………………………..……..……..14

| UAS-mcd GFP | | | |
| --- | --- | --- | --- |
| 40B | **42B** | **HL9** | **R5802** |
| n=6 | n=6 | n=6 | n=4 |
| 44.83 ±5.26 | 41.00 ±1.37 | 60.10 ±07.54* | 101 ±3.43 |

Supplementary Table 1 . PAM dopamine cell counts per hemisphere. *HL9 numbers from Claridge-Chang et al. 2009 (Claridge-Chang et al., 2009)

|  |  | | Odor1 | |  | |  | | Odor 2 | |  | |  |
| --- | --- | --- | --- | --- | --- | --- | --- | --- | --- | --- | --- | --- | --- |
| GAL4 lines | **+/GAL4** | | **+/UAS** | | **GAL4/UAS** | | **+/GAL4** | | **+/UAS** | | **GAL4/UAS** | |  |
| R58E02 | 0.73 ±0.05 | | 0.83 ±0.06 | | 0.92 ±0.04 | | 0.36 ±0.05 | | 0.51 ±0.11 | | 0.66 ±0.07 | |  |
| HL9 | 0.53 ±0.11 | | 0.49 ±0.08 | | 0.62 ±0.08 | | 0.49 ±0.10 | | 0.44 ±0.13 | | 0.33 ±0.07 | |  |
| MB109B | 0.71 ±0.06 | | 0.68 ±0.05 | | 0.80 ±0.04 | | 0.37 ±0.05 | | 0.67 ±0.03 | | 0.68 ±0.06 | |  |
| MB058B | 0.69 ±0.04 | | 0.78 ±0.04 | | 0.90 ±0.02 | | 0.56 ±0.04 | | 0.57 ±0.04 | | 0.67 ±0.03 | |  |
| MB399B | 0.67 ±0.06 | | 0.62 ±0.05 | | 0.92 ±0.02 | | 0.49 ±0.05 | | 0.59 ±0.03 | | 0.67 ±0.04 | |  |
| MB018B | | 0.65 ±0.06 | | 0.74 ±0.06 | | 0.57 ±0.04 | | 0.47 ±0.07 | | 0.41 ±0.09 | | 0.48 ±0.04 | |

Supplementary Table 2. Odor Controls at 30C. Odor 1: Isoamyl Acetate or Ethyl Acetate. Odor 2: Isoamyl Alcohol. Naïve flies were presented with either odor 1 vs air or odor 2 vs air in the Y maze.

| **Split-GAL4 lines** | **Target Expression Pattern with Intensity** | **Off Target Expression** |
| --- | --- | --- |
| **MB040B** | PAM-α1 (2), PAM-β`1ap (2), PAM-β`1m (2), PAM-β`2a (1), PAM-β`2m (2), PAM-β`2p (2), PAM-β2 (3), PAM-γ3 (3), PAM-γ4 (3), PAM-γ5 (3) | Diffuse central brain expression |
| **MB042B** | PAM-α1(1), PAM-β`1ap (1), PAM-β`1m (1), PAM-β`2a (1), PAM-β`2m (2), PAM-β`2p (1), PAM-β1 (1), PAM-γ3 (3), PAM-γ4 (3), PAM-γ4<γ1γ2 (1), PAM-γ5 (3) | Limited posterior VNC expression with reporter: pJFRC206-5xUAS-IVS-myr::smGFP-FLAG in VK00005 |
| **MB188B** | PAM-β`1ap (5), PAM-β`1m (5), PAM-γ3 (3), PAM-γ4 (2) | Limited posterior VNC expression with reporter: pJFRC206-5xUAS-IVS-myr::smGFP-FLAG in VK00005 |
| **MB032B** | PAM-β’2m (4), PAM-β`2p (1), PAM-β2β`2a (1), PAM-γ3 (1) | Limited central brain expression with reporter: pJFRC2-10xUAS-IVS-mCD8::GFP in VK00005 |
| **MB301B** | PAM-β’2m (1), PAM-β2β`2a (3) | VNC expression with reporter: pJFRC200-10XUAS-IVS-myr::smGFP-HA in attP18 |
| **MB109B** | PAM-β`2a (5), PAM-γ5 (1) | Limited posterior VNC expression with reporter: pJFRC206-5xUAS-IVS-myr::smGFP-FLAG in VK00005 |
| **MB315C** | PAM-γ5 (5) | Limited posterior VNC expression with reporter: pJFRC206-5xUAS-IVS-myr::smGFP-FLAG in VK00005 |
| **MB299B** | PAM-α1 (3), PAM-β1 (1), PAM-β2 (1) | Limited posterior VNC expression with reporter: pJFRC206-5xUAS-IVS-myr::smGFP-FLAG in VK00005 |
| **MB210B** | MBON-β`2mp (4), MBON-β`2mp_bilateral (1), MBON-γ5β`2a (5) | Limited posterior VNC expression with reporters: 20xUAS-IVS-CsChrimson-mVenus in attP18 pJFRC2-10xUAS-IVS-mCD8::GFP in VK00005  Posterior VNC expression with reporter: pJFRC206-5xUAS-IVS-myr::smGFP-FLAG in VK00005 |
| **MB011B** | MBON-β`2mp (3), MBON-β`2mp_bilateral (3), MBON- γ5β`2a (4) | Limited central brain expression with reporter: pJFRC2-10xUAS-IVS-mCD8::GFP in VK00005  Limited posterior VNC expression with reporter: pJFRC206-5xUAS-IVS-myr::smGFP-FLAG in VK00005 |
| **MB399B** | MBON-β2β`2a (2) | Limited posterior VNC expression with reporter: pJFRC2-10xUAS-IVS-mCD8::GFP in VK00005  Central brain expression with reporter: pJFRC206-5xUAS-IVS-myr::smGFP-FLAG in VK00005 |
| **MB074C** | MBON-β`2mp (4), MBON-β2β`2a (3), MBON- γ5β`2a (1) | Limited posterior VNC expression with reporter: pJFRC206-5xUAS-IVS-myr::smGFP-FLAG in VK00005 |
| **MB002B** | MBON-β`2mp (4), MBON- γ5β`2a (2) | Limited posterior VNC expression reporter: pJFRC206-5xUAS-IVS-myr::smGFP-FLAG in VK00005 |

Supplementary Table 3. Summary of all target and off target expression of each split-GAL4 line used in the paper as described on FlyLight (<https://www.janelia.org/project-team/flylight>). Intensity of expression reported as low (1) to high (5).

| **Figure** | **Experiment** | **n** | **Statistical Test** | **Result** | **Post-hoc** | **Result** |
| --- | --- | --- | --- | --- | --- | --- |
| 1B | Acquisition | +/R58E02 (n=23)  +/shi^ts^ (n=23)  shi^ts^/R58E02 (n=23) | One-way ANOVA | F(2, 66)=5.355, p=0.007 | Tukey | +/ shi^ts^ vs +/R58E02 p=0.87  +/ shi^ts^ vs shi^ts^/R58E02 p=0.009  +/R58E02 vs shi^ts^/R58E02 p=0.04 |
| 1B | Consolidation | +/R58E02 (n=13)  +/shi^ts^ (n=15)  shi^ts^/R58E02 (n=13) | One-way ANOVA | F(2,38)=5.964, p=0.00559 | Tukey | +/ shi^ts^ vs +/R58E02 p=0.004  +/ shi^ts^ vs shi^ts^/R58E02 p=0.18  +/R58E02 vs shi^ts^/R58E02 p=0.26 |
| 1B | Retrieval | +/R58E02 (n=25)  +/shi^ts^ (n=24)  shi^ts^/R58E02 (n=25) | One-way ANOVA | F(2,71)=5.707, p=0.005 | Tukey | +/ shi^ts^ vs +/R58E02 p=0.65  +/ shi^ts^ vs shi^ts^/R58E02 p=0.05  +/R58E02 vs shi^ts^/R58E02 p=0.005 |
| 1C | D2R | +/R58E02 (n=37)  +/D2Ri (n=37)  D2Ri/R58E02 (n=37) | One-way ANOVA | F(2,89)=6.441, p=0.002 | Tukey | +/ D2Ri vs +/R58E02 p=0.980  +/D2Ri vs D2Ri/R58E02 p=0.0099  +/R58E02 vs D2Ri/R58E02 p=0.0056 |
| 1F | Calcium Imaging  Early Epoch | GCaMP6m/R58E02 (n=6) | Repeated Measures ANOVA | F(1,5)=8.705, p=0.03 | N/A | N/A |
| 1G | Calcium Imaging  Late Epoch | GCaMP6m/R58E02 (n=6) | Repeated Measures ANOVA | F(1,5)=24,177, p=0.004 | N/A | N/A |
| 2A | Retrieval | +/40B (n=7)  +/shi^ts^ (n=6)  shi^ts^/40B (n=7) | One-way ANOVA | F(2,17)=2.43, p=0.12 | N/A | N/A |
| 2B | Retrieval | +/42B (n=16)  +/shi^ts^ (n=16)  shi^ts^/42B (n=14) | One-way ANOVA | F(2,68)=0.995, p=0.38 | N/A | N/A |
| 2C | Retrieval | +/188B (n=24)  +/shi^ts^ (n=27)  shi^ts^/188B (n=25) | One-way ANOVA | F(2,73)=0.044, p=0.96 | N/A | N/A |
| 2D | Retrieval | +/32B (n=20)  +/shi^ts^ (n=20)  shi^ts^/32B (n=20) | One-way ANOVA | F(2,57)=1.164, p=0.32 | N/A | N/A |
| 2E | Retrieval | +/301B (n=23)  +/shi^ts^ (n=24)  shi^ts^/301B (n=24) | One-way ANOVA | F(2,78)=0.389, p=0.68 | N/A | N/A |
| 2F | Retrieval | +/109B (n=20)  +/shi^ts^ (n=24)  shi^ts^/109B (n=24) | One-way ANOVA | F(2,65)=14.18, p= 7.78x10^-6 | Tukey | +/ shi^ts^ vs +/MB109B p=0.07  +/ shi^ts^ vs shi^ts^/MB109B p=0.007  +/MB109B vs shi^ts^/MB109B p=0.000005 |
| 2G | Retrieval | +/315C (n=20)  +/shi^ts^ (n=19)  shi^ts^/315C (n=20) | One-way ANOVA | F(2,56)=0.109, p=0.90 | N/A | N/A |
| 2H | Retrieval | +/299B (n=9)  +/shi^ts^ (n=13)  shi^ts^/MB299B (n=13) | One-way ANOVA | F(2,32)=1.468, p=0.246 | N/A | N/A |
| 3A | Acquisition | +/2B (n=16)  +/shi^ts^ (n=17)  shi^ts^/2B (n=17) | One-way ANOVA | F(2,47)=0.31, p=0.73 | N/A | N/A |
| 3B | Acquisition | +/210B (n=26)  +/shi^ts^ (n=26)  shi^ts^/210B (n=27) | One-way ANOVA | F(2,76)=1.59, p=0.21 | N/A | N/A |
| 3C | Acquisition | +/11B (n=17)  +/shi^ts^ (n=15)  shi^ts^/11B (n=15) | One-way ANOVA | F(2,44)=0.09, p=0.92 | N/A | N/A |
| 3D | Acquisition | +/399B (n=25)  +/shi^ts^ (n=26)  shi^ts^/399B (n=25) | One-way ANOVA | F(2,73)=0.90, p=0.42 | N/A | N/A |
| 3E | Acquisition | +/74C (n=11)  +/shi^ts^ (n=12)  shi^ts^/74C (n=12) | One-way ANOVA | F(2,32)=0.30, p=0.75 | Tukey | N/A |
| 3F | Retrieval | +/2B (n=19)  +/shi^ts^ (n=19)  shi^ts^/2B (n=19) | One-way ANOVA | F(2,54)=2.05, p=0.14 | N/A | N/A |
| 3G | Retrieval | +/210B (n=29)  +/shi^ts^ (n=29)  shi^ts^/210B (n=26) | One-way ANOVA | F(2,81)=0.52, p=0.60 | N/A | N/A |
| 3H | Retrieval | +/11B (n=19)  +/shi^ts^ (n=19)  shi^ts^/11B (n=18) | One-way ANOVA | F(2,53)=0.40, p=0.67 | N/A | N/A |
| 3I | Retrieval | +/399B (n=22)  +/shi^ts^ (n=19)  shi^ts^/399B (n=21) | One-way ANOVA | F(2,59)=5.62, p=0.006 | Tukey | +/ shi^ts^ vs +/MB399B p=0.93  +/ shi^ts^ vs shi^ts^/MB399B p=0.010  +/MB399B vs shi^ts^/MB399B p=0.02 |
| 3J | Retrieval | +/MB074C (n=32)  +/shi^ts^ (n=30)  shi^ts^/MB074C (n=32) | One-way ANOVA | F(2,91)=2.22, p=0.11 | Tukey | N/A |
| 4C | Consolidation | +/MB002B (n=20)  +/shi^ts^ (n=18)  shi^ts^/MB002B (n=19)  +/MB074C (n=21)  +/shi^ts^ (n=21)  shi^ts^/MB074C (n=21) | One-way ANOVA | F(2,54)= 9.287, p=0.0003  F(2,71)= 3.51, p=0.04 | Tukey  Tukey | +/ shi^ts^ vs +/MB002B p=0.989  +/ shi^ts^ vs shi^ts^/MB002B p=0.001  +/MB002B vs shi^ts^/MB002B p=0.001  +/ shi^ts^ vs +/MB074C p=0.46  +/MB074C vs shi^ts^/MB074C p=0.14  +/ shi^ts^ vs shi^ts^/MB074C p=0.008 |
| 4D | D2R | +/MB002B (n=22)  +/D2Ri (n=22)  D2Ri/MB002B (n=22)  +/MB074C (n=26)  +/D2Ri (n=23)  D2Ri/MB074C (n=25) | One-way ANOVA | F(2,63)=12.77, p=2.22x10^-05  F(2,71)=3.51, p=0.04 | Tukey  Tukey | +/ D2Ri vs +/MB002B p=0.07  +/ D2Ri vs D2Ri/MB002B p=0.00001  +/MB002B vs D2Ri/MB002B p=0.019  +/ D2Ri vs +/74C p=0.47  +/ D2Ri vs D2Ri/74C p=0.03  +/74C vs D2Ri/74C p=0.29 |
| 5C | Acquisition | +/MB018B (n=20)  +/shi^ts^ (n=21)  shi^ts^/MB018B (n=25) | One-way ANOVA | F(2,63)=2.18, p=0.12 | N/A | N/A |
| 5D | Retrieval | +/MB018B (n=36)  +/shi^ts^ (n=38)  shi^ts^/MB018B (n=45) | One-way ANOVA | F(2,116)=19.46, p=5.17x10^-08 | Tukey | +/ shi^ts^ vs +/MB018B p=0.40  +/ shi^ts^ vs shi^ts^/MB018B p=0.00004  +/18B vs shi^ts^/18B p=0.0000001 |
| 5E | Acquisition | +/MB058B (n=28)  +/shi^ts^ (n=30)  shi^ts^/MB058B (n=30) | One-way ANOVA | F(2,85)=0.202, p=0.817 | N/A | N/A |
| 5F | Retrieval | +/MB058B (n=18)  +/shi^ts^ (n=20)  shi^ts^/MB958B (n=22) | One-way ANOVA | F(2,54)=5.103, p=0.009 | Tukey | +/ shi^ts^ vs +/MB058B p=0.81  +/ shi^ts^ vs shi^ts^/MB058B p=0.049  +/MB058B vs shi^ts^/MB058B p=0.013 |
| F1.S1 | Group Activity | +/R58E02 (n=15)  +/shi^ts^ (n=15)  shi^ts^/R58E02 (n=15) | Repeated Measures ANOVA  Mauchly’s test | F(3.38, 76)= 16.21, p=0.00  χ^2^(5)=90.51, p=0.00; ε=0.563 | Bonferonni | Baseline:  +/ shi^ts^ vs +/R58E02 p=1.00  +/ shi^ts^ vs shi^ts^/R58E02 p=0.00  +/R58E02 vs shi^ts^/R58E02 p=0.00  Early EtOH:  +/ shi^ts^ vs +/R58E02 p=0.00  +/ shi^ts^ vs shi^ts^/R58E02 p=0.232  +/R58E02 vs shi^ts^/R58E02 p=0.002  Late EtOH:  +/ shi^ts^ vs +/R58E02 p=0.00  +/ shi^ts^ vs shi^ts^/R58E02 p=0.016  +/R58E02 vs shi^ts^/R58E02 p=0.380  Recovery:  +/ shi^ts^ vs +/R58E02 p=0.127  +/ shi^ts^ vs shi^ts^/R58E02 p=0.075  +/R58E02 vs shi^ts^/R58E02 p=1.000 |
| F1.S2 | Dopamine Fluorescence | Air (n=7)  Ethanol (n=11) | One-way ANOVA | F(1,16)=2.947, p=0.105 | N/A | N/A |
| F1.S3D | Calcium Imaging Early Epoch Odor 1 vs 2 | GCaMP6m/R58E02 (n=6) | F(1,5)=0.144, p=0.720 | N/A | N/A | N/A |
| F1.S3G | Calcium Imaging Late Epoch Odor 1 vs 2 | GCaMP6m/R58E02 (n=6) | F(1,5)=3.437, p=0.123 | N/A | N/A | N/A |
| F1.S3K | Calcium Imaging  Early Ethanol vs Late Ethanol | GCaMP6m/R58E02 (n=6) | F(1,5)=0.390, p=0.560 | N/A | N/A | N/A |
| F1.S4A | Dopamine Acquisition and Retrieval | shi^ts^/MB042B (n=11)  shi^ts^/MB196B (n=11)  shi^ts^/MB299B (n=6)  shi^ts^/MB047B (n=11)  shi^ts^/MB195B (n=12)  shi^ts^/MB316B (n=12)  shi^ts^/MB312B (n=10)  shi^ts^/MB194B (n=12)  shi^ts^/MB025B (n=11)  shi^ts^/MB043B (n=11)  shi^ts^/MB213B (n=11)  shi^ts^/MB301B (n=12)  shi^ts^/MB040B (n=11)  shi^ts^/MB087C (n=12)  shi^ts^/MB315C (n=12)  shi^ts^/MB109B (n=12)  shi^ts^/MB188B (n=10)  shi^ts^/MB032B (n=11)  shi^ts^/PBP (n=11) | Kruskal-Wallis | χ^2^(18)=30.81, p=0.03 | Dunnett’s Test | shi^ts^/MB042B vs shi^ts^/PBP p=0.38  shi^ts^/MB196B vs shi^ts^/PBP p=0.64  shi^ts^/MB299B vs shi^ts^/PBP p=0.55  shi^ts^/MB047B vs shi^ts^/PBP p=0.71  shi^ts^/MB195B vs shi^ts^/PBP p=0.79  shi^ts^/MB316B vs shi^ts^/PBP p=0.71  shi^ts^/MB312B vs shi^ts^/PBP p=0.49  shi^ts^/MB194B vs shi^ts^/PBP p=0.71  shi^ts^/MB025B vs shi^ts^/PBP p=0.17  shi^ts^/MB043B vs shi^ts^/PBP p=0.86  shi^ts^/MB213B vs shi^ts^/PBP p=0.67  shi^ts^/MB301B vs shi^ts^/PBP p=0.27  shi^ts^/MB040B vs shi^ts^/PBP p=0.54  shi^ts^/MB087C vs shi^ts^/PBP p=0.17  shi^ts^/MB315C vs shi^ts^/PBP p=0.04  shi^ts^/MB109B vs shi^ts^/PBP p=0.03  shi^ts^/MB188B vs shi^ts^/PBP p=0.008  shi^ts^/MB032B vs shi^ts^/PBP p=0.01 |
| F1.S4B | MB Acquisition and Retrieval | shi^ts^/MB010B (n=10)  shi^ts^/MB152B (n=12)  shi^ts^/MB364B (n=11)  shi^ts^/MB009B (n=12)  shi^ts^/MB417B (n=11)  shi^ts^/MB005B (n=12)  shi^ts^/MB370B (n=12)  shi^ts^/MB461B (n=12)  shi^ts^/MB008B (n=11)  shi^ts^/MB371B (n=12)  shi^ts^/PBP (n=12) | Kruskal-Wallis | χ^2^(10)=27.97, p=0.002 | Dunnett’s Test | shi^ts^/MB010B vs shi^ts^/PBP p=0.04  shi^ts^/MB152B vs shi^ts^/PBP p=3.69x10^-05  shi^ts^/MB364B vs shi^ts^/PBP p=0.04  shi^ts^/MB009B vs shi^ts^/PBP p=4.86x10^-04  shi^ts^/MB417B vs shi^ts^/PBP p=2.68x10^-04  shi^ts^/MB005B vs shi^ts^/PBP p=0.002  shi^ts^/MB370B vs shi^ts^/PBP p=0.04  shi^ts^/MB461B vs shi^ts^/PBP p=0.04  shi^ts^/MB008B vs shi^ts^/PBP p=1.87x10^-04  shi^ts^/MB371B vs shi^ts^/PBP p=0.045 |
| F1.S6A | Acquisition | +/HL9 (n=8)  +/shit^s^ (n=8)  shi^ts^ /HL9 (n=8) | One-way ANOVA | F(2,21)=0.24, p=0.788 | N/A | N/A |
| F1.S6B | Consolidation | +/HL9 (n=8)  +/shi^ts^ (n=8)  shi^ts^/HL9 (n=8) | One-way ANOVA | F(2,21)=0.698, p=0.509 | N/A | N/A |
| F1.S6C | Retrieval | +/HL9 (n=8)  +/shi^ts^ (n=8)  shi^ts^ /HL9 (n=8) | One-way ANOVA | F(2,21)=8.596, p=0.00187 | Tukey | +/ shi^ts^ vs +/HL9 p=0.92  +/ shi^ts^ vs shi^ts^/HL9 p=0.003  +/HL9 vs shi^ts^/HL9 p=0.007 |
| F1.S6A | Acquisition | +/MB040B (n=23)  +/shi^ts^ (n=23)  shi^ts^/MB040B (n=21) | One-way ANOVA | F(2,64)=1.262, p=0.39 | N/A | N/A |
| F1.S6B | Acquisition | +/MB042B (n=24)  +/shi^ts^ (n=24)  shi^ts^/MB042B (n=23) | One-way ANOVA | F(2,68)=0.995, p=0.38 | N/A | N/A |
| F1.S6C | Acquisition | +/MB188B (n=10)  +/shi^ts^ (n=11)  shi^ts^/MB188B (n=12) | One-way ANOVA | F(2,30)=0.084, p=0.92 | N/A | N/A |
| F1.S6D | Acquisition | +/MB032B (n=22)  +/shi^ts^ (n=21)  shi^ts^/MB032B (n=20) | One-way ANOVA | F(2,60)=1.52, p=0.23 | N/A | N/A |
| F1.S6E | Acquisition | +/MB301B (n=27)  +/shi^ts^ (n=27)  shi^ts^/MB301B (n=27) | One-way ANOVA | F(2,78)=0.389, p=0.68 | N/A | N/A |
| F1.S6F | Acquisition | +/MB109B (n=24)  +/shi^ts^ (n=24)  shi^ts^/MB109B (n=24) | One-way ANOVA | F(2,69)=0.091, p=0.91 | N/A | N/A |
| F1.S6G | Acquisition | +/315C (n=20)  +/shi^ts^ (n=23)  shi^ts^/315C (n=24) | One-way ANOVA | F(2,64)=0.24, p=0.79 | N/A | N/A |
| F1.S6H | Acquisition | +/299B (n=20)  +/shi^ts^ (n=23)  shi^ts^/299B (n=24) | One-way ANOVA | F(2,31)=0.6, p=0.555 | N/A | N/A |
| F1.S7 | mRNA PCR | D1R1/elav (n=6)  +/D1R1 (n=6)  D1R2/elav (n=6)  +/D1R2 (n=6)  D2R/elav (n=3)  +/D2R (n=3) | One-way ANOVA | F(1,10)=20.05, p=0.001  F(1,10)=30.31, p=0.0003  F(1,4)=19.14, p=0.011 | N/A | N/A |
| F1.S8A | R58E02 Temperature Controls | +/R58E02 (n=15)  +/ shi^ts^ (n=15)  shi^ts^/R58E02 (n=15) | One-way ANOVA | F(2,42)=1.953, p=0.155 | N/A | N/A |
| F1.S8B | HL9 Temperature Controls | +/HL9 (n=8)  +/ shi^ts^ (n=8)  shi^ts^/HL9 (n=8) | One-way ANOVA | F(2,21)=0.823, p=0.453 | N/A | N/A |
| F1.S8C | MB109B Temperature Controls | +/MB109B (n=35)  +/MB109B (n=35)  shi^ts^/MB109B (n=35) | One-way ANOVA | F(2,102)=0.411, p=0.664 | N/A | N/A |
| F4.S7B | D2R | +/MB399B (n=23)  +/dD2R (n=23)  dD2R/MB399B (n=23) | One-way ANOVA | F(2,65)=0.032, p=0.968 | N/A | N/A |
| F4.S7C | D1R1;D1R2 | +/MB399B (n=14)  +/dD1R1;dD1R2 (n=15)  dD1R1;dD1R2/MB399B (n=15) | One-way ANOVA | F(2,41)=0.223, p=0.801 | N/A | N/A |
| F4.S7D | D1R1i;D1R2i | +/MB074C (n=26)  +/D1R1;D1R2 (n=28)  D1R1;D1R2/MB074C (n=28) | One- way ANOVA | F(2,79)=0.123, p=0.884 | N/A | N/A |
| F4.S8 | 399B Temperature Controls | +/399B (n=18)  +/shit^s^ (n=18)  shi^ts^/399B (n=18) | One-way ANOVA | F(2,51)=1.039, p=0.361 | N/A | N/A |
| F5.S1A | D1R1;D1R2 | +/MB018B (n=14)  +/D1R1i;D1R2i (n=14)  D1R1iD1R2i/MB018B (n=12) | One-way ANOVA | F(2,37)=2.00, p=0.15 | N/A | N/A |
| F5.S1B | D2R | +/MB018B (n=20)  +/D2Ri (n=20)  D2Ri/MB018B (n=20) | One-way ANOVA | F(2,57)=0.113, p=0.90 | N/A | N/A |
| F5.S2A | 18B Temperature Controls | +/18B (n=24)  +/shit^s^ (n=25)  shi^ts^/18B (n=25) | One-way ANOVA | F(2,71)=0.225, p=0.799 | N/A | N/A |
| F5.S2B | MB058B Temperature Controls | +/MB058B (n=18)  +/MB058B (n=18)  shi^ts^/MB058B (n=18) | One-way ANOVA | F(2,50)=0.516, p=0.6 | N/A | N/A |
|  |  |  |  |  |  |  |

Supplementary Table 4. Statistical Analysis Summary for Main Figures and Supplemental Figures

| Target Gene | Stock # | Citation |
| --- | --- | --- |
| *Dop1R1* | VDRC-KK-107058 | (Wang et al., 2016, Wang et al., 2013, Lark et al., 2017, Ferguson et al., 2017, Agrawal and Hasan, 2015) |
| *Dop1R2* | VDRC-GD-3391 | (Wang et al., 2016, Regna et al., 2016, Dietzl et al., 2007) |
| *D2R* | VDRC-GD-11471 | (Andreatta et al., 2018, Bang et al., 2011, Neckameyer and White, 1993, Petruccelli et al., 2018, Wang et al., 2016, Dietzl et al., 2007, Agrawal and Hasan, 2015, Shang et al., 2011) |

Supplementary Table 5. Previous publications using RNAi lines in this paper.

**Supplemental References**

AGRAWAL, T. & HASAN, G. 2015. Maturation of a central brain flight circuit in Drosophila requires Fz2/Ca(2)(+) signaling. *Elife,* 4.

ANDREATTA, G., KYRIACOU, C. P., FLATT, T. & COSTA, R. 2018. Aminergic Signaling Controls Ovarian Dormancy in Drosophila. *Sci Rep,* 8**,** 2030.

BANG, S., HYUN, S., HONG, S. T., KANG, J., JEONG, K., PARK, J. J., CHOE, J. & CHUNG, J. 2011. Dopamine signalling in mushroom bodies regulates temperature-preference behaviour in Drosophila. *PLoS Genet,* 7**,** e1001346.

CLARIDGE-CHANG, A., ROORDA, R. D., VRONTOU, E., SJULSON, L., LI, H., HIRSH, J. & MIESENBOCK, G. 2009. Writing memories with light-addressable reinforcement circuitry. *Cell,* 139**,** 405-15.

DIETZL, G., CHEN, D., SCHNORRER, F., SU, K. C., BARINOVA, Y., FELLNER, M., GASSER, B., KINSEY, K., OPPEL, S., SCHEIBLAUER, S., COUTO, A., MARRA, V., KELEMAN, K. & DICKSON, B. J. 2007. A genome-wide transgenic RNAi library for conditional gene inactivation in Drosophila. *Nature,* 448**,** 151-6.

FERGUSON, L., PETTY, A., ROHRSCHEIB, C., TROUP, M., KIRSZENBLAT, L., EYLES, D. W. & VAN SWINDEREN, B. 2017. Transient Dysregulation of Dopamine Signaling in a Developing Drosophila Arousal Circuit Permanently Impairs Behavioral Responsiveness in Adults. *Front Psychiatry,* 8**,** 22.

LARK, A., KITAMOTO, T. & MARTIN, J. R. 2017. Modulation of neuronal activity in the Drosophila mushroom body by DopEcR, a unique dual receptor for ecdysone and dopamine. *Biochim Biophys Acta Mol Cell Res,* 1864**,** 1578-1588.

NECKAMEYER, W. S. & WHITE, K. 1993. Drosophila tyrosine hydroxylase is encoded by the pale locus. *J Neurogenet,* 8**,** 189-99.

PETRUCCELLI, E., FEYDER, M., LEDRU, N., JAQUES, Y., ANDERSON, E. & KAUN, K. R. 2018. Alcohol Activates Scabrous-Notch to Influence Associated Memories. *Neuron,* 100**,** 1209-1223 e4.

REGNA, K., KURSHAN, P. T., HARWOOD, B. N., JENKINS, A. M., LAI, C. Q., MUSKAVITCH, M. A., KOPIN, A. S. & DRAPER, I. 2016. A critical role for the Drosophila dopamine D1-like receptor Dop1R2 at the onset of metamorphosis. *BMC Dev Biol,* 16**,** 15.

SHANG, Y., HAYNES, P., PIREZ, N., HARRINGTON, K. I., GUO, F., POLLACK, J., HONG, P., GRIFFITH, L. C. & ROSBASH, M. 2011. Imaging analysis of clock neurons reveals light buffers the wake-promoting effect of dopamine. *Nat Neurosci,* 14**,** 889-95.

WANG, Q. P., LIN, Y. Q., ZHANG, L., WILSON, Y. A., OYSTON, L. J., COTTERELL, J., QI, Y., KHUONG, T. M., BAKHSHI, N., PLANCHENAULT, Y., BROWMAN, D. T., LAU, M. T., COLE, T. A., WONG, A. C., SIMPSON, S. J., COLE, A. R., PENNINGER, J. M., HERZOG, H. & NEELY, G. G. 2016. Sucralose Promotes Food Intake through NPY and a Neuronal Fasting Response. *Cell Metab,* 24**,** 75-90.

WANG, Y., PU, Y. & SHEN, P. 2013. Neuropeptide-gated perception of appetitive olfactory inputs in Drosophila larvae. *Cell Rep,* 3**,** 820-30.
